# Interlocking intramedullary nail for forearm diaphyseal fractures in adults—A systematic review and meta-analysis of outcomes and complications

**DOI:** 10.1186/s10195-024-00761-7

**Published:** 2024-04-13

**Authors:** Ali Lari, Yousef Hassan, Abdulwahab Altammar, Ali Esmaeil, Abdulaziz Altammar, Carlos Prada, Ali Jarragh

**Affiliations:** 1 Department of Orthopedic Surgery, AlRazi National Orthopedic Hospital, Kuwait City, Kuwait; 2 St Joseph’s Health Care, Hand and Upper Limb Centre, University of Western Ontario, London, ON Canada; 3 Department of Orthopedic Surgery, Kuwait University, Kuwait City, Kuwait

**Keywords:** Intramedullary nail, Interlocking nail, Forearm fractures, Radius, Nlna, Diaphysis

## Abstract

**Purpose:**

The purpose of this systematic review is to examine the outcomes, complications, and potential advantages of using anatomical interlocking intramedullary nails (IMN) in the treatment of radius and ulnar shaft diaphyseal fractures in adults.

**Methods:**

Medline, Embase, Web of Science, and Cumulative Index to Nursing and Allied Health Literature (CINAHL) databases were searched between January 2000 and January 2023. Studies meeting criteria were observational or randomized controlled trials evaluating outcomes in IMN for adult diaphyseal forearm fractures. Standardized data extraction was performed and a quality assessment tool was used to evaluate individual study methodology. Descriptive statistics for interventions, functional outcomes, and complications were reported. Meta-analysis was performed for patient-reported outcome measures and operative time.

**Results:**

A total of 29 studies involving 1268 patients were included with 764 (60%) undergoing IMN, 21% open reduction and internal fixation (ORIF), and 9% hybrid fixation. There was no significant difference between groups in DASH and Grace–Eversmann scores. Operative time was significantly shorter in IMN compared with ORIF. The DASH scores were: 13.1 ± 6.04 for IMN, 10.17 ± 3.98 for ORIF, and 15.5 ± 0.63 in hybrids. Mean operative time was 65.3 ± 28.7 in ORIF and 50.8 ± 17.7 in IMN. Complication rates were 16.7% in the IMN group, 14.9% in ORIF, and 6.3% in hybrid constructs. There were 11 cases of extensor pollicis rupture in the IMN group. Average IMN pronation and supination were 78.3° ± 7.9° and 73° ± 5.0°, respectively. Average ORIF pronation and supination was 82.15° ± 1.9° and 79.7° ± 4.5°, respectively.

**Conclusions:**

Similar functional outcomes and complication rates along with shorter operative times can be achieved with IMN compared with ORIF. The use of IMN is promising, however, higher quality evidence is required to assess appropriate indications, subtle differences in range of motion, implant-related complications, and cost-effectiveness.

*Trail Registration* PROSPERO (International Prospective Register of Systematic Reviews) (ID: CRD42022362353).

*Level of evidence* III.

## Introduction

Forearm fractures involving the diaphysis of the radius and ulna hold a distinct place in orthopedic injuries due to their frequency and the anatomical importance of the involved bones’ distinct capacity to perform pronation and supination [1]. The curvature of the radius, with its pronounced bow, is essential for maintaining the alignment and range of motion of the proximal and distal radioulnar joints [2]. Thus, orthopedic surgeons approach the radial bow with the reverence reserved for joints aiming to achieve anatomical reduction when treating these fractures.

For adult patients, open reduction and internal fixation (ORIF) through separate surgical approaches is currently considered the gold standard in the management of these fractures, allowing for an anatomic restoration of the fractures [3]. While often successful, open reduction is associated with an often-generous surgical approach, periosteal stripping, and soft tissue damage. Non-locking intramedullary nailing has been the predominant method of fixation in the pediatric population due to the nature of pediatric bone healing in growth and remodeling potential [4]. In adults, the use of Kirschner wires, Steinmann pins, and Rush rods yielded an unacceptably high failure rate in the form of nonunion and malrotation [5]. Therefore, efforts were put into the development of anatomical interlocking IMNs. Often, the nails are pre-contoured to the shape of the radius or ulna in addition to being locked proximally or distally by use of fluoroscopy to aid in the insertion of locking screws.

However, as is often the case with emerging medical technologies, while the advantages of these anatomical IMN seem promising, comprehensive data evaluating their efficacy, advantages, and potential complications are still in the nascent stages, and there has not been a widespread adoption of them to treat forearm fractures. There are suggestions in the orthopedic community that these nails might be particularly beneficial in specific challenging scenarios, such as open fractures, extensive soft tissue injuries, or cases involving hemodynamically unstable patients [6].

The purpose of this systematic review is to examine the outcomes, complications, and potential advantages of using anatomical IMNs in the treatment of radius and ulnar shaft diaphyseal fractures. We aim to provide data on the current state of this treatment modality to help guide future research and clinical decision-making.

## Methods

The search and selection process followed the Preferred Reporting Items for Systematic Reviews and Meta-Analyses (PRISMA) guidelines and was prospectively registered with PROSPERO (International Prospective Register of Systematic Reviews) (ID: CRD42022362353).

### Search strategy

A systematic search of Medline, Embase, Web of Science, and Cumulative Index to Nursing and Allied Health Literature (CINAHL) databases between January 2000 and January 2023 was performed using the following keywords: (forearm) OR (radius) OR (ulna) OR (radial) OR (ulnar) AND (Nail) OR (intramedullary) OR (Hybrid). Finally, reference lists of relevant articles were reviewed to identify additional articles that were potentially missed during the initial search.

### Eligibility criteria

The inclusion criteria for the studies were as follows: The studies must have used interlocking intramedullary nails (IMN) for the treatment of acute open or closed diaphyseal fractures of the radius and/or ulna. The patient population had to be aged 18 years or older. Additionally, the studies needed to provide clinical outcomes, such as union rates, functional outcomes, and complications related to the use of interlocking IMN.

The exclusion criteria included: review articles, as they do not present original research data; studies without full text availability; cadaveric or biomechanical studies; studies treating pediatric patients, defined as individuals under the age of 18 years; the use of flexible or non-locking intramedullary nails; studies including the treatment of forearm septic or aseptic nonunions; fractures involving the proximal and distal joints; pathological fractures; and ipsilateral upper limb fractures, including those of the humerus and wrist, as these could confound the outcomes related to the forearm fractures. In addition, studies that did not report the nail design were excluded.

### Study screening

Titles and abstracts were independently screened for relevance by three authors using Covidence (Covidence systematic review software, Veritas Health Innovation, Melbourne, Australia. www.covidence.org). Potentially relevant articles underwent full-text screening, with any conflicts between the authors being resolved by discussion and consensus with the senior authors.

### Quality assessment and risk of bias

Study quality assessment was conducted using the methodological index for non-randomized studies (MINORS) tool. Methodological quality was categorized prior as follows: a score of 0–8 or 0–12 was considered poor quality, 9–12 or 13–18 was considered fair quality, and 13–16 or 19–24 was considered excellent quality, for noncomparative and comparative studies, respectively. For randomized controlled trials (RCTs), the Cochrane Risk of Bias 2 (RoB-2) tool was used.

### Data extraction

Three authors independently extracted relevant data from the included studies to a previously piloted Microsoft Excel spreadsheet (Microsoft, Redmond, Washington, USA). These data included general article information, patient demographic and surgical procedure details, and relevant outcome measures.

### Data analysis

Descriptive statistics including the mean, range and measures of variance [e.g., standard deviations, 95% confidence intervals (CI)] were utilized where applicable. Data were synthesized into pooled demographics, treatment, and outcome measures. Measures of spread were calculated from each study if individual data were reported. The mean was used to summarize continuous outcomes in all studies. However, the minimum and maximum values were reported along with the mean in some studies. In these cases, the range was used to estimate the standard deviation.

### Effect size calculation

Meta-analysis was performed using R v. 4.3. For continuous variables, the mean difference (MD) between groups was used as the measure of effect size for the relative ease of interpretation compared with the standardized mean difference. For categorical variables, the odds ratio (OR) was calculated for each study and a meta-analysis of OR was performed. The unadjusted OR was calculated using the provided counts and total numbers within each group. We selected the odds ratio as the preferred measure of effect size for categorical variables because it remains unaffected by variations in the baseline probability of the control group, which can differ among studies. The analysis was performed for the percentage of patients with excellent and good scores combined.

### Meta-analysis

Statistical analysis was performed using R v. 4.3. The 95% confidence interval (CI) and *Z*-statistic were calculated and used for hypothesis testing the generic inverse method for weighting. Between-study statistical heterogeneity was assessed using the random-effects heterogeneity parameter tau, and *I*^2^ statistics (defined as the percentage of variability in effect estimates due to statistical heterogeneity rather than sampling error) were generated for all meta-analyses. Given the expected variation in research design and participant characteristics, all meta-analyses used a random-effects model with restricted maximum likelihood (REML) to quantify between-study heterogeneity.

### Sensitivity analysis

Sensitivity analysis was performed, using the leave-one-out method, to assess the effect of the different studies on the estimate (OR) and heterogeneity. Sensitivity analysis was performed to assess whether the pooled estimate and between-study heterogeneity were significantly affected by the exclusion of certain studies.

### Publication bias and heterogeneity between studies

Funnel plots were used to assess publication bias. Egger’s test was used to test the asymmetry of funnel plots [7]. The trim-and-fill method was also used to detect and adjust for publication bias [8]. We used the method suggested by Pustejovsky and Rodgers when testing for the funnel plot asymmetry, as the effect is dependent on the standard error [9].

## Results

### Sample data

**Fig. 1:**
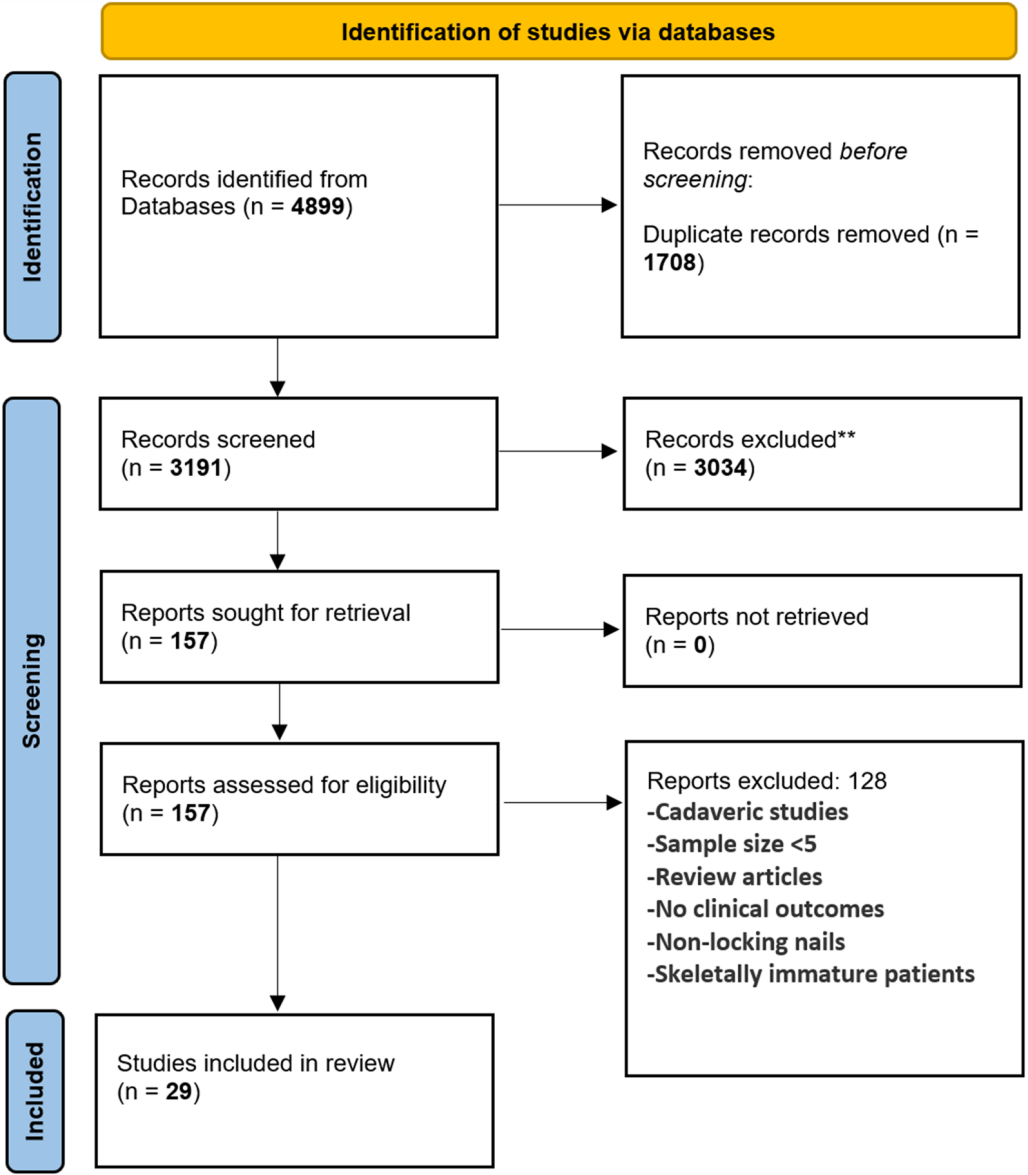
PRISMA flow diagram summarizing the study selection process

The study designs and demographics of the included studies are summarized in Table 1. A total of 29 studies were included (Fig. 1); 14 were retrospective cohorts, 8 were retrospective comparative studies, 5 were prospective cohorts, and 2 were randomized controlled trials (RCT). A total of 11 studies (38%) compared ORIF with IMN. A total of 1268 patients were included, with male predominance (74%).

**Table 1:** Demographics of the sample data and characteristics of the included studies

Study	Country	Study type	Minors	Sample size	Female, *N* (%)	Age, mean (years)	Follow-up mean (months)	AO classification
Weißer 2003 [29]	Germany	P. cohort	15	32	9 (28%)	36.7	16.1	NR
Gao 2005 [24]	China	R. cohort	12	18	4 (22.2%)	46	13	A (16%) B (55%) C (27%)
Weckbach 2006 [25]	Germany	P. cohort	15	32	9 (28.12%)	36.7	31.4	A (55%) B (32%) C (13%)
Visńa 2008 [30]	Czech republic	R. cohort	12	78	28 (35.89%)	37.02	25	A (37.1) B (38.4%) C (24.3)
Lee 2008 [6]	South Korea	R. cohort	12	27	NR	32	17	A (32%) B (50%) C (18%)
Ozkaya 2009 [31]	Turkey	Case control	11	42	13 (31%)	32.5	26.5	NR
Bansal 2011 [32]	India	P. cohort	15	12	3 (25%)	32	28	A (33%) B (50%) C (16%)
Behnke 2012 [12]	Canada	Case control	14	56	16	31.7	16.5	NR
Lil 2012 [33]	India	P. cohort	16	34	10	38.1	18	NR
Saka 2013 [34]	Turkey	R. cohort	11	18	5 (28%)	28	24 (12–36)	A (60%) B (35%), C (1%)
Lee 2014 [15]	South Korea	RCT	*Some concerns	67	22 (33%)	41	20 (18–65)	A (45%) B (55%)
Saka 2014 [35]	Turkey	R. cohort	12	23	6 (26%)	34	27 (12–42)	A (83%) B (17%)
Saka 2014 [26]	Turkey	R. cohort	12	43	5 (12%)	37	28 (12–44)	A (72%) B (28%)
Köse 2014 [36]	Turkey	R. cohort	10	18	5 (27%)	35	17.7	A (44%) B (44%) C (11%)
Kim 2015 [13]	South Korea	Case control	13	47	17(36%)	47.3	16.2	A (36%) B (29%) C (35%)
Zhang 2016 [10]	China	RCT	*Some concerns	87	40 (46%)	38.03	23.4 (12–26)	NR
Al-Sadek 2016 [37]	UAE	Case control	12	45	NR	NR	NR	2R2, 2U2
Köse 2016 [38]	Turkey	R. cohort	12	17	6 (35.3%)	35.7	38 (36–52)	A (76%) B (24%)
Babu 2017 [39]	India	R. cohort	12	68	NR	NR	5 (2–8)	NR
Kose 2017 [17]	Turkey	Case control	14	90	42 (47%)	37	15	A (36.6) B (33.3%) C (30%)
Azboy 2017 [20]	Turkey	R. cohort	11	32	9 (28.1%)	36	17(13–28)	A (37.5%) B (40.6) C (21.8%)
Yörükoğlu 2017 [28]	Turkey	R. cohort	12	23	6 (26%)	38.6	31 ± 16	A (39.1%) B (47.8%) C (13%)
Kibar 2019 [16]	Turkey	Case control	14	57	19 (33.3%)	39.1	25 (12–60)	A (63.2%) B (29.8%) C (7%)
Lee 2019 [40]	South Korea	Case control	14	101	35 (34.7%)	42	34 (24–67)	A (48%) B (51%)
Kibar 2020 [14]	Turkey	R. cohort	12	49	10 (20.4%)	35	26 (12–48)	A (73%) B (24%) C (2%)
Uygur 2021 [41]	Turkey	R. cohort	12	78	25 (32%)	33.4	26.4 (12–46)	2R2 2U2
Pavone 2021 [11]	Italy	Case control	15	23	7 (30.4%)	46.4	12	2U2
Kale 2021 [19]	India	P. cohort	16	30	7 (23%)	33.5	NR	A (76.6%) B (13.3%) C (10%)
Blažević 2021 [18]	Croatia	R. cohort	12	21	6 (28%)	38	NR	A (71.4%) B (19%) C (9.5%)
Aggregate values				Total: 1268	% female 26.6%	Mean 36.97	Mean 22.41 SD 7.55	AO: A 384/B 274/C 104 Fracture: BB 810/R 243/U 272

*R. cohort* retrospective cohort, *P. cohort* prospective cohort, *RCT* randomized controlled trial, *NR* not reported, *AO* Association of Osteosynthesis, *BB* Bone bone, *R* Radius, *U* Ulna

### Interventions characteristics

The interventions and implants used are detailed in Table 2. Six studies utilized the ForeSight IMN, six used the Acumed forearm rod system, and ten used the locking nail by TST (Rakor Medical Instruments Industry, Turkey). A total of 764 patients (60%) had IMN, 275 (21%) were treated by ORIF with plating, and 111 (8.7%) were treated with hybrid constructs. Postoperative regimens exhibited variability; 11 studies did not enforce immobilization after nailing, whereas 9 studies employed variable immobilization approaches.

**Table 2:** Implant types, interventions, and immobilization regimens for the included studies

Study	Implant	Intervention	Immobilization	Open fracture, *N* (%)
Weißer 2003 [29]	Smith & Nephew: foresight	IMN	NR	NR
Gao 2005 [24]	Smith & Nephew: foresight	IMN	2–3 weeks	8 (44%)
Weckbach 2006 [25]	Smith & Nephew: foresight	IMN	None	5 (15%)
Visńa 2008 [30]	Smith & Nephew: foresight	IMN	Selective immobilization	20 (25.3%)
Lee 2008 [6]	Acumed: rod system	IMN	6 weeks	7 (18%)
Ozkaya 2009 [31]	NR: ILN	ORIF 22/IMN 20	ORIF: none/IMN: 2–3 weeks	3 (7.1%): ORIF: 2 (9.1%) IMN: 1 (5%)
Bansal 2011 [32]	NR: square nail	IMN	None	3 (25%)
Behnke 2012 [12]	Smith & Nephew: foresight	ORIF 27/hybrid 29	1 week	10 (17.9%)
Lil 2012 [33]	Talwalkar: square nail	IMN	Immobilization (NR)	2 (7.4%)
Saka 2013 [34]	TST: ILN	IMN	None	2 (11%)
Lee 2014 [15]	Acumed: rod system	ORIF 32/IMN 35	ORIF: none IMN: 2 weeks	19 (28%): ORIF: 10/32 (31%) IMN: 9/35 (26%)
Saka 2014 [35]	TST: ILN	IMN	None	3 (13%)
Saka 2014 [26]	TST: ILN	IMN	None	2 (5%)
Köse 2014 [36]	TST: ILN	IMN	2–3 days	NR
Kim 2015 [13]	Acumed: rod system	ORIF 31 hybrid 16	2–3 weeks	16 (34%)
Zhang 2016 [10]	Smith & Nephew: foresight	ORIF 21 IMN 22 hybrid 44	2 weeks	0
Al-Sadek 2016 [37]	Talwalkar: square nail	23 ORIF 22 IMN	Immobilization (NR)	0
Köse 2016 [38]	TST: ILN	IMN	None	0
Babu 2017 [39]	Talwalkar: square nail	IMN	6 weeks	0
Kose 2017 [17]	TST: ILN	ORIF 42 IMN 48	IMN: none / ORIF: immobilized	IMN 12 (13%) ORIF 8 (9%)
Azboy 2017 [20]	TST: ILN	IMN	Immobilize: type C	9 (28%)
Yörükoğlu 2017 [28]	Acumed: rod system	IMN	NR	0
Kibar 2019 [16]	TST: ILN	ORIF 30 IMN 27	ORIF: 2–3 weeks / IMN: none	ORIF: 2 (6.7%) IMN: 2 (7%)
Lee 2019 [40]	Acumed: rod system	ORIF 41 IMN 28 hybrid 32	ORIF: none IMN: 2 weeks	ORIF 12 (30%) IMN 8 (28%) hybrid 8 (25%)
Kibar 2020 [14]	TST: ILN	ORIF 22 IMN 27	ORIF: 2–3 weeks IMN: none	5 (10.2%)
Uygur 2021 [41]	TST: ILN	IMN	2 weeks	23 (29.4%)
Pavone 2021 [11]	Acumed: rod system	ORIF 14 IMN 9	ORIF: 2 weeks IMN: none	0
Kale 2021 [19]	NR: ILN	IMN	6 days	0
Blažević 2021 [18]	Treu-instrumente GmbH nail	IMN	None	1 (5%)
Aggregate		Total IMN: 764 Total ORIF: 275 Total hybrid: 111		Total open fractures: 190 (16.5%) Open fracture ORIF: 34 (12.3%) Open fracture IMN/hybrid: 170 (13.5%)

*ORIF* open reduction internal fixation, *IMN* intramedullary nail, *ILN* interlocking nail

### Surgical outcomes

#### Patient-reported outcome measures (PROMs)

Patient-reported outcome measures (PROMs) and range of motion (ROM) values are illustrated in Table 3. The DASH score was utilized in 22 studies, yielding average scores of 13.1 ± 6.0, 10.2 ± 3.9, and 15.6 ± 0.6 for IMN, ORIF, and hybrid constructs, respectively. The Grace–Eversmann (GE) score was employed in 15 studies, revealing distribution of outcomes within the IMN group as follows: 70.7% excellent, 21.5% good, 3.9% acceptable, and 3.7% poor. In the ORIF group, corresponding percentages were 76% excellent, 11.6% good, 8.4% acceptable, and 10.4% poor.

**Table 3:** Functional outcomes, range of motion, and operative time for the included studies

Study	Patient-reported outcome measures (PROM) mean (range/SD)				Range of motion (ROM) forearm-pronation supination					OR time (mean minutes)
Weißer 2003 [29]	DASH 11.3				NR					67
Gao 2005 [24]	DASH 19 (4–72)				P 62 S 80					78
Weckbach 2006 [25]	DASH 13.7				NR					67
Visńa 2008 [30]	Anderson score: full ROM 89%, restricted 11%				P 62 S 80					75
Lee 2008 [6]	DASH 15 (5–61)	GES EX (22, 81%) GD (3, 11%) AC (2, 7%)			P 85 (82–89) S 87 (83–90)					45
Ozkaya 2009 [31]	DASH ORIF: 15 (4–30) IMN: 13 (3–25)	GES ORIF: EX (14, 63%), GD (4, 18%), AC (4, 18%) IMN: EX (16, 80%), GD (2, 10%), AC (2, 10%)			NR					65
Bansal 2011 [32]	DASH: 14 (8–36)	GE: EX (11, 87%) GD (1, 12%)			11 full ROM/1 limited ROM					35
Behnke 2012 [12]	GE: ORIF EX (18, 66%) GD (2, 7.4%), AC (5, 18.5%), P (2, 7.4%) hybrid EX (17, 58%) GD (5, 17%), AC (6, 20%), P (1, 3%)				ORIF: P 82.2 ± 16.5 (30–90) S 78.5 ± 22.2 (10–90) hybrid: P 84.5 ± 13.2 (30–90) S 79.1 ± 20.2 (20–90)					NR
Lil 2012 [33]	DASH: 15 (4–36)	GE: EX (17, 50%) GD (10, 29%) AC (4, 11%) P (3, 9%)			NR					42
Saka 2013 [34]	DASH: 8.08 (0–17.5)	GE: EX (15, 83%), GD (2, 11%), P (1, 5%)			P 80 (70–90) S 82 (80–90)					NR
Lee 2014 [15]	DASH: ORIF: 15 ± 3 IMN: 18 ± 3	GE: ORIF: EX (19, 86%), GD (2, 9%), P (1, 4%) IMN: EX (21, 84%), GD (3, 12%), AC (1, 4%)			ORIF PS arc 159 (154–164) IMN PS arc 157 (151–163)					ORIF: 74 ± 8 IMN: 52 ± 10
Saka 2014 [35]	DASH: 4.2 (0–13.3)	GE: EX or GD (21, 91%) AC (2, 9%)			P 84 S 82					20 (15–32)
Saka 2014 [26]	DASH: 6.5 (0–13.3)	GE: EX (38, 88%), GD (5, 12%)			P 80 S 82					30 (23–45)
Köse 2014 [36]	DASH: 15.15 (4–38.8)				P 83 S 73					61.94
Kim 2015 [13]	DASH: ORIF: 7.1 hybrid: 15.1				ORIF: P 79 (50–90) S 85 (70–90) hybrid: P 73 (60–90) S 76 (50–90)					NR
Zhang 2016 [10]	Anderson’s scoring system: ORIF: EX (11, 52.4%) GD (3, 14.3%) P (7, 33.3%) IMN: EX (11, 50%) GD (5, 22.7%) P (6, 27%) hybrid: EX (34, 77%) GD (5, 11%) P (5, 11%)									ORIF: 137 IMN: 77 Hybrid: 68
Al-Sadek 2016 [37]	Anderson’s scoring system: ORIF: EX (20, 87%) GD (3, 13%) IMN: Ex (15, 68%) GD (3, 13.9%) P (4, 18%)									NR
Köse 2016 [38]	DASH 12.58 (3.3–32.5)	GE All Ex, 1 GD			P 85 (74–90) S 75 (67–80)					27.5 (17–70)
Babu 2017 [39]	NR				NR					NR
Kose 2017 [17]	DASH: ORIF 9.81 (3.3–30) IMN 12.87 (3.3–38.8)	GE IMN EX (40, 83%), GD (8, 16%) ORIF EX (36, 85%), GD (4, 9%), AC (1, 2%), P (1, 2%)			IMN P 84 (74–90) S 75 (65–80)			ORIF P 84 (64–90) S 74 (65–80)		ORIF: 63 IMN: 46
Azboy 2017 [20]	Dash 14 (5–36)	GE: EX (21, 65%), GD (7, 22%), AC (3, 9%), P (1, 3%)								52
Yörükoğlu 2017 [28]	DASH 31.2 ± 24.9				P 72.2 ± 19.8 S 75.3 ± 24.7					NR
Kibar 2019 [16]	DASH ORIF: 7.7 ± 8.6 IMN: 7 ± 4.5	GE: ORIF: EX (24, 80.0%), GD (2, 6.7%), AC (4, 13.3%) IMN: EX (19, 70.4%), GD (7, 25.9%), AC (1,3.7%)			ORIF: S 81.7 ± 5.9° P 81.7 ± 6.5		IMN: S 82.6 ± 4.5° P 80.7 ± 8.3°			ORIF: 63 IMN 46
Lee 2019 [40]	DASH: ORIF: 15 ± 3 IMN: 18 ± 3 hybrid: 16 ± 4	GE: ORIF: EX (35, 85.3%) GD (4, 9.7%) AC (2, 4.9%) IMN: EX (23, 82.1%) GD (3, 10.7%) AC (2, 7.1%) Hybrid: EX (27, 84.4%) GD (3, 9.4%) AC (2, 6.2%)			Pronation-supination arc ORIF: 161 ± 5 IMN: 156 ± 6 hybrid: 160 ± 2					ORIF: 74 IMN: 59 Hybrid: 68
Kibar 2020 [14]	DASH: ORIF: 6.56 ± 9.9 IMN: 4.42 ± 3.96	GE: ORIF: EX (13, 59.1%) GD (5, 22.7%) AC (4, 18.2%) IMN: EX (21, 77.8%) GD (4, 14.8%) AC (2, 7.4%)			ORIF: P 83.41 ± 5.21 S 81.59 ± 5.21 IMN: P 84.81 ± 4.27 S 83.04 ± 4.66					IMN 21 (15–35) ORIF 46 (40–110)
Uygur 2021 [41]	Dash: 10.5 (0–56)	GE: EX/GD (75, 96%), P (3, 4%)			NR					NR
Pavone 2021 [11]	GE: EX (22, 73%) GD (4, 13%) AC (2, 7%) P (2, 7%)				P 75 (50–80) S 68 (50–80)					NR
Kale 2021 [19]	NR				3 Patients: 30 °C P–S impairment compared with CLS					NR
Blažević 2021 [18]	DASH ORIF (5.21 ± 2.21) IMN (4.68 ± 1.66)				NR					NR
Aggregate mean values	DASH ORIF: 10.17 ± 3.98 IMN: 13.1 ± 6.04 Hybrid: 15.5 ± 0.63	GE ORIF EX (190, 76%) GD (29, 11.6%) AC (21, 8.4%) P (10, 4%)	GE IMN EX (377, 70.7%) GD (115, 21.5%) AC (21, 3.9%) P (20, 3.75%)	GE hybrid EX (78, 74.2%) GD (13, 12.3%) AC (8, 7.6%) P (6, 5.7%)	IMN pronation: 78.3 ± 7.89 Supination: 73 ± 4.99	ORIF pronation 82.15 ± 1.93 Supination 79.7 ± 4.47			Hybrid pronation 78.78 ± 5.75 Supination 77.55 ± 1.55	Surgical time ORIF 65.3 ± 28.7 IMN: 50.8 ± 17.7 Hybrid 68

*DASH* disabilities of the arm, shoulder and hand score, *GE* Grace–Eversmann score, *OR* operative time, *PS* pro-supination, *P* pronation, *S* supination. Grace–Eversmann Score: *EX* excellent, *GD* good, *AC* acceptable, *P* poor

#### Range of motion (ROM)

Pronation and supination data were available from 17 studies. In the IMN group, the average aggregate pronation was 78.3 ± 7.9, and supination was 73 ± 5.0. For the ORIF group, pronation measured 82.15 ± 1.9, while supination was 79.7 ± 4.5. In hybrid constructs, pronation and supination values were 78.8 ± 5.7 and 77.5 ± 1.5, respectively.

### Complications and reinterventions

The complications and reoperations are outlined in Table 4. The overall complication rate was 16.7% (*n* = 128) in the IMN group, 14.9% (*n* = 41) in ORIF, and 6.3% (*n* = 7) in hybrid constructs. Within the IMN group, complications included delayed union (3.5%) (*n* = 27), infection (3.1%) (*n* = 24), and nonunion (2.2%) (*n* = 17). Notably, there were 11 cases (1.4%) of extensor pollicis longus ruptures in the IMN group, whereas none were reported in ORIF. Nerve palsies were encountered in eight patients (0.93%) in IMN and three patients (1%) in ORIF. All nerve palsies were successfully managed through conservative measures.

**Table 4:** Complications and reoperations for the included studies

Study	Complications, *N* (%)	Nonunion, *N* (%)	Delayed union, *N* (%)	Infection, *N* (%)	Synostosis, *N* (%)	Others	Implant removal, *N* (%)	Non-routine reinterventions, *N* (%)
Weißer 2003 [29]	4 (20%)	0	2 (6.3%)	0	1 complete 1 incomplete	0	13 (40.6%)	1 (3%) implant removal and resection of synostosis
Gao 2005 [24]	7 (22%)	0	0	4 (12.5%) sup	1	2 screw loosening	0	0
Weckbach 2006 [25]	6 (19%)	1 (3%)	2 (6%)	0	2 (6%)	1 fracture distraction	19 (59%) (routine)	2 synostosis resection 1 nonunion revision
Visńa 2008 [30]	15 (19%)	0	4 (6%)	1 (1.2%) sup	3 incomplete	1 compartment synd 6 distal screw migration (7.2%)	27 (35%) (routine)	1 fasciotomy
Lee 2008 [6]	2 (7.4%)	1 (3.5%)	0	1 (3.5%) sup	0	0	5 (13%) (routine)	0
Ozkaya 2009 [31]	ORIF: 3 (13%) IMN: 2 (10%)	0	0	ORIF: 3 (13%) sup	0	0	ORIF: 12 (54.5%) (routine) IMN: 7 (35%)	0
Bansal 2011 [32]	2 (16%)	0 (0%)	1 (8.3%)	1 (8.3%) sup	0	0	3 (25%)	0
Behnke 2012 [12]	ORIF: 6 (10%) IMN: 4 (7%)	ORIF: 1 (1.7%) IMN: 1 (1.7%)	0	0	ORIF: 1 (1.7%)	ORIF: 1 CRPS, 2 RNP, 1 refracture IMN: 2 EPL rupture, 1 RNP	0	ORIF: 1 nonunion revision IMN: 1 nonunion revision 1 EPL rupture reconstruction
Lil 2012 [33]	7 (21%)	3 (9%)	0	2 (6%) sup	1	1 olecranon bursitis	0	3 revision nonunion
Saka 2013 [34]	3 (16.6%)	0	1 (5.5%)	2 (11%) Sup	0	0	1 (5.5%)	1 (5%) revision nonunion
Lee 2014 [15]	ORIF:1(3.1%) IMN: 3 (8.6%)	IMN: 1 (2.9%)	IMN: 2 (5.7%)	ORIF: 1 (3.1%) sup	0	0	IMN: 1 (2.9%)	IMN: 1 (2.9%) revision nonunion
Saka 2014 [35]	0	0	0	0	0	0	0	0
Saka 2014 [26]	2 (4.6%)	0	0	2 (4.6%) sup	0	0	0	0
Köse 2014 [36]	1 (5.7%)	0	0	o		1 (5.7%) EPL rupture	0	1 EPL reconstruction
Kim 2015 [13]	ORIF: 1 (3%) Hybrid: 2(12%)	ORIF: 1 (3%) Hybrid: 2 (12%)	0	0	0	0	ORIF: 1 Hybrid: 2	ORIF: 1 (3%) revision nonunion Hybrid: 2 (12%) revision nonunion
Zhang 2016 [10]	ORIF: 6 (28%) IMN: 6 (27%) Hybrid: 5 (25%)	ORIF 3 (14.3%)	0	ORIF: 3 (3.4%) Hybrid: 1 (4.3%)	0	IMN: 6 (27%) 3 malunion (13.6%) 3 nerve palsy (13.6%) Hybrid: 4 (19%) 2 malunion (9.5%) 2 nerve palsy (9.5%)	0	0
Al-Sadek 2016 [37]	ORIF: 1 (4.3%) IMN: 5 (22.7%)	IMN 2 (9%)	IMN: 2 (9%)	ORIF: 1 (4.3%) sup	0	IMN: 1 (4.5%) malunion	0	2 revision nonunion
Köse 2016 [38]	0	0	0	0	0	0	0	0
Babu 2017 [39]	24 (35%)	0	8 (12%) Sup	8 (12%)	0	3 (6%) nail migration	0	0
Kose 2017 [17]	IMN: 2 (2%) ORIF: 3 (3%)	IMN: 0 ORIF: 1	0	IMN: 1 sup ORIF: 2 sup	0	IMN: 1 (1%) EPL rupture	IMN: 4 (4%) routine Plate 4 (4%) routine	ORIF: 1 revision nonunion IMN: 1 EPL reconstruction
Azboy 2017 [20]	IMN: 8 (25%)	0 (0%)	1 (3.1%)	2 (6.3%) Sup	1 (3.1%)	0	4 (12.5%)	1 (3.1%) synostosis excision
Yörükoğlu 2017 [28]	IMN: 11 (47%)	3 (13.1)	0	0	2 (8.6%)	EPL rupture 6 (26.8)	0	0
Kibar 2019 [16]	ORIF: 4 (13.3%)	ORIF: 1 (3.33%)	0	ORIF: 2 (6.7%) sup	0	0	ORIF: 1 (3.3%)	ORIF: 1 revision nonunion
Lee 2019 [40]	ORIF: 2 (4.9%) IMN: 3 (10.7%)	IMN: 1 (3.6%)	IMN: 2 (7.1%)	ORIF: 1 (2.4%) sup	0	0	ORIF: 1 (2.4%)	ORIF: 1 (2.4%) refracture IMN: 1 (3.6%) nonunion
Kibar 2020 [14]	ORIF: 5 (22.7%)	ORIF: 1 (4.5%)	0	ORIF: 2 (9%) Sup	0	ORIF: 1 (4.5%) PIN neuropraxia	ORIF: 2 (9.1%)	ORIF: 2 (9.1%) -Revision nonunion -Symptomatic implant
Uygur 2021 [41]	IMN: 3 (3.9%)	0 (0%)	2 (2.6%)	0 (0%)	1 (1.3%)	0	0	0
Pavone 2021 [11]	ORIF: 9 (64%)	0	ORIF: 3 (21%)	ORIF: 1 (7%) sup	0	ORIF: 5 (35%) persistent pain	0	0
Kale 2021 [19]	4 (13%)	0	0	0	0	1 PIN palsy (3%) 3 swelling (10%)	0	0
Blažević 2021 [18]	3 (14%)	1(4%)	0	0	0	1 RNP (4%) 1 EPL rupture (4%)	0	1 revision nonunion 1 EPL reconstruction
Aggregate values	ORIF: 41 (14.9%) IMN: 128 (16.7%) Hybrid: 7 (6.3%)	ORIF: 8 (2.9%) IMN: 17 (2.2%) Hybrid: 2 (1.8%)	ORIF: 3 (1%) IMN: 27 (3.5%) Hybrid: 0	ORIF:16 (5.8%) IMN: 24 (3.1%) Hybrid: 1 (0.9%)	ORIF: 1 (0.36%) IMN: complete 8 (1%) Incomplete: 5 (0.65%) Hybrid: 0	Mechanical IMN: 11 (1.4%) Malunion IMN: 4 (0.52%) Hybrid: 2 (1.8%) Tendon injury IMN: 11 (1.4%) Nerve injury ORIF: 3 (1%) IMN: 8 (1%)	ORIF: 21 (7.6%) IMN: 84 (10.9%) Hybrid: 2 (1.8%)	ORIF: 7 (1.8%) IMN: 20 (2.6%) Hybrid: 2 (1.8%)

*sup* superficial, *CRPS* complex regional pain syndrome, *RNP* radial nerve palsy, *EPL* extensor pollicis longus, *PIN* posterior interosseous nerve

Non-routine reoperation rates were 2.6%, 1.8%, and 1.8% for the IMN, ORIF, and hybrid groups, respectively. Within the IMN group, most non-routine reoperations involved addressing extensor pollicis longus injuries (1.8%, *n* = 11), nonunion revisions (2.2%, *n* = 17), and excision of complete synostosis (0.93%, *n* = 8).

### Comparative studies

In an RCT, Lee et al. found that ORIF resulted in a significantly shorter time to radiographic union compared with IMN (10 ± 3 versus 14 ± 5 weeks). In addition, significantly shorter fluoroscopic exposure time was reported in ORIF versus IMN (2.0 ± 0.7 versus 7.0 ± 3.0 min, respectively). Although ORIF more accurately restored the radial bow, functional outcomes were similar between the two methods. Notably, female patients receiving IMN reported significantly higher satisfaction levels. Range of motion did not differ significantly across both groups (ORIF: 159 ± 5, IMN: 157 ± 6, *p* = 0.55).

In a separate RCT, Zhang et al. observed that IMN led to significantly reduced operative time, smaller periosteal stripping area, and a smaller incision size than ORIF (p < 0.01) [10]. Moreover, hybrid fixation, which employed a radius plate and ulna nail, yielded the best functional results with the fewest complications (*p* = 0.03). ORIF presented more cases of nonunions, while IMN and hybrid fixation had more malunions.

In retrospective comparisons, Pavone et al. identified shorter times to radiographic union with IMN than ORIF. They also highlighted a quicker return to work or sports for IMN patients, averaging 2.3 months, as opposed to 5.8 months for ORIF (*p* < 0.01) [11]. Behnke et al., on the contrary, found no significant difference in union time between ORIF and hybrid constructs, with comparable complication rates [12]. Conversely, Kim et al. reported superior outcomes in terms of ROM, union time, and PROMs with ORIF-only treatments for both bone fractures when compared with hybrid constructs [13] Fig. 1.

## Meta-analysis

### Comparison of DASH score between IMN and ORIF

**Fig. 2:**
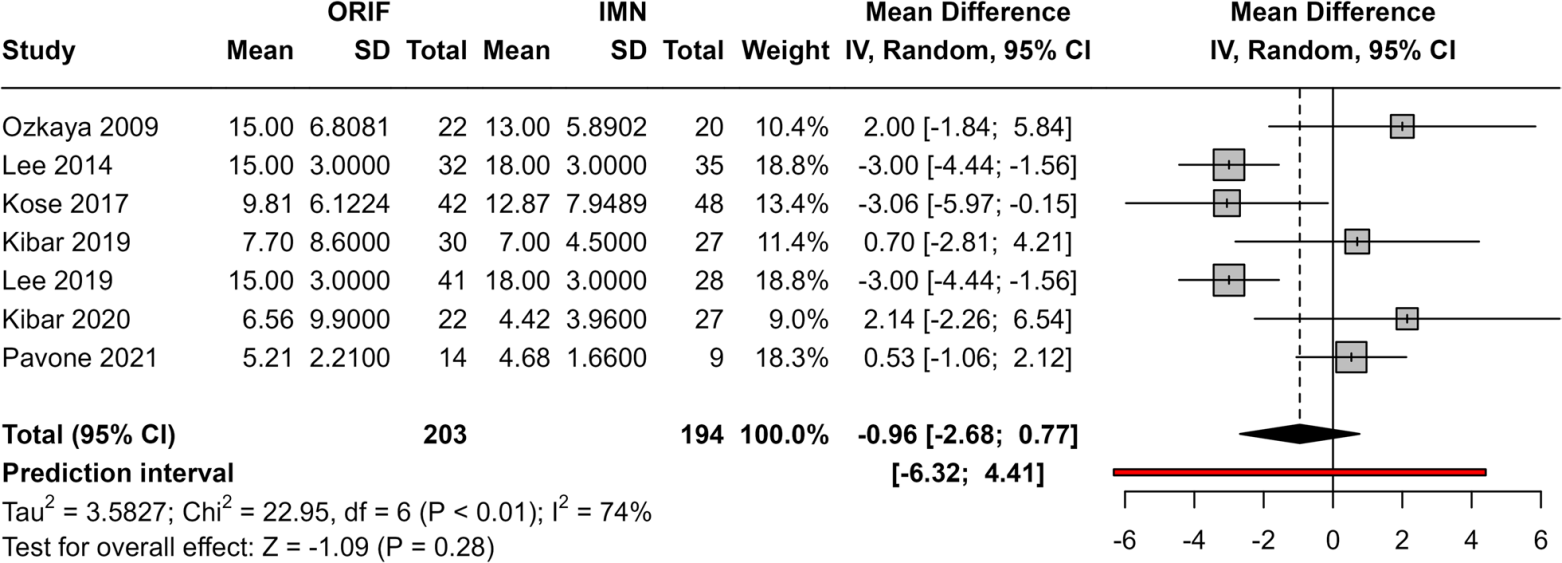
Meta-analysis for mean difference in DASH scores between groups

A total of seven studies reported the average DASH score in the ORIF and IMN groups. There was no significant difference in the pooled mean difference between groups (MD = −0.96, 95% CI −2.68; 0.77, *p* = 0.28) (Fig. 2). Substantial heterogeneity was observed between studies (*I*^2^ = 74%, *p* < 0.01) that was not explained by the exclusion of any of the included studies. The funnel plot was symmetric suggesting the absence of publication bias. Egger’s test was not performed due to the low number of studies (*n* < 7).

### Comparison of Grace–Eversmann score between IMN and ORIF

**Fig. 3:**
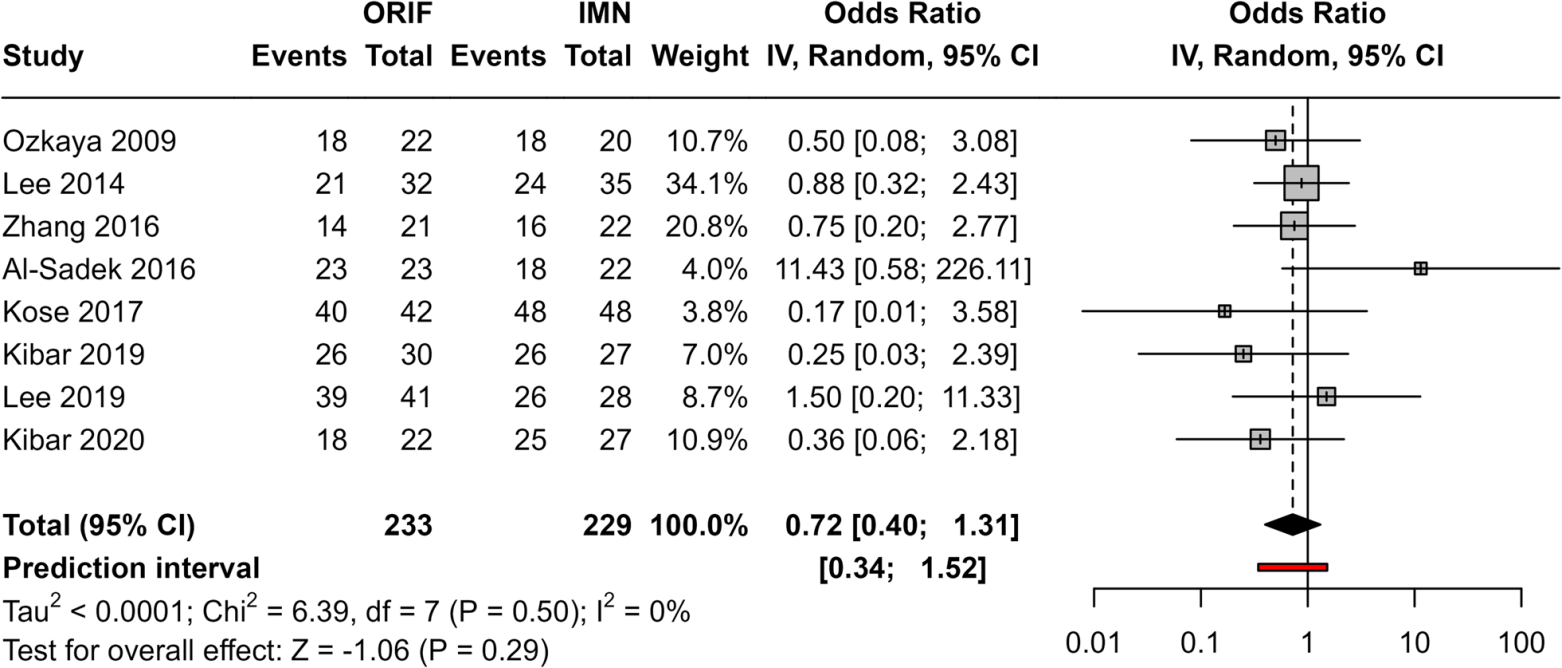
Meta-analysis of odds ratio for excellent/good GE score in ORIF patients compared with IMN patients

The meta-analysis included eight studies (Fig. 3). The pooled odds ratio was not statistically significant (OR 0.72, 95% CI 0.4, 1.31; *p* = 0.29) with no heterogeneity observed between studies (*I*^2^ = 0%, *p* = 0.5). The lack of association persisted when the trim-and-fill method was used.

### Comparison of operative time between IMN and ORIF

**Fig. 4:**
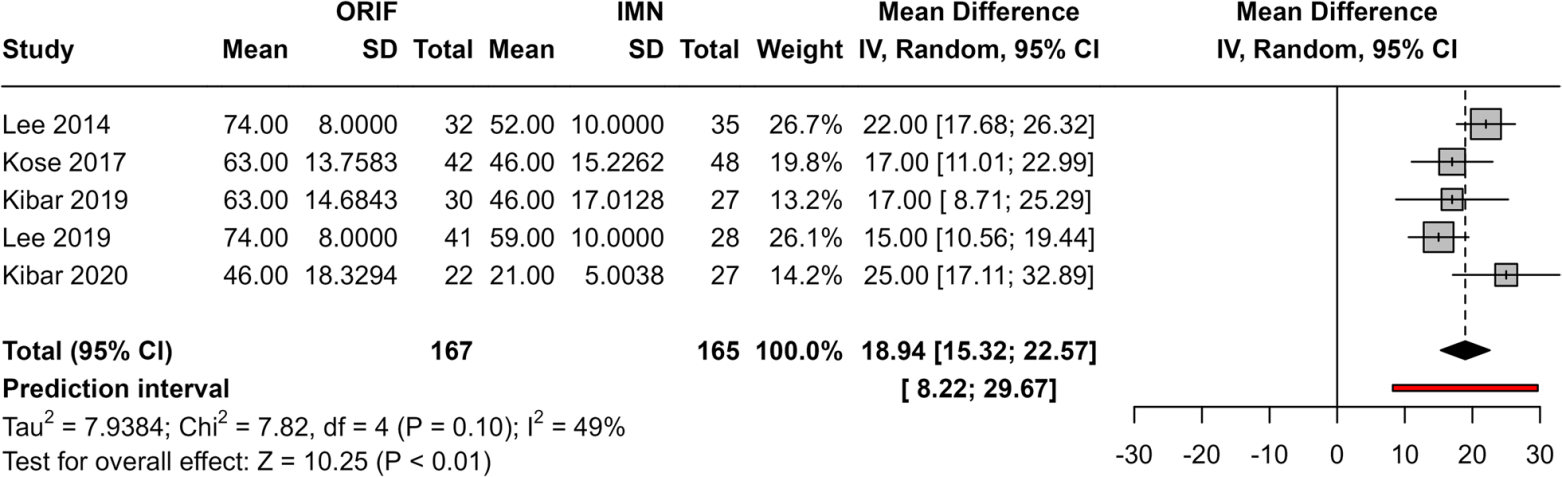
Meta-analysis for mean difference in operative time between groups

The meta-analysis included five studies. Results showed that the pooled mean difference in the operative time was significantly higher in the ORIF group than the IMN group (Fig. 4.) (MD 18.94, 95% CI 15.32, 22.57; *p* < 0.01). Moderate heterogeneity was observed between studies (*I*^2^ = 49%, *p* = 0.1) although statistical testing (tau^2^) revealed that such heterogeneity is not statistically significant.

## Discussion

The management of diaphyseal forearm fractures has undergone evolutions over the years, with ORIF being the gold standard for adult patients. However, the search for a less invasive technique and the favorable results with long bone IMN treatment has led to the advent and application of the anatomical interlocking IMN systems. Our systematic review’s main finding suggests that clinical outcomes of IMN are satisfactory and comparable to ORIF with plates, achieving similar complication rates (16.7% versus 14.9%).

There were a number of implants used in the studies, all unified by an anatomical design and interlocking screws to ensure rotation stability. The overall patient reported outcome measures were favorable for both IMN and ORIF with no statistically significant difference (DASH: 10.1 ± 4 versus 13.1 ± 6) (*p* = 0.28). In addition, both techniques achieved functional arcs of motion, with aggregate values that were higher for ORIF than IMN. Due to the inconsistent reporting of range of motion, a subgroup analysis was not feasible to determine whether there was a significant difference between groups. While this finding may suggest that patients undergoing ORIF are more likely to recover a full arc of motion, firm conclusions cannot be drawn unless fracture types are matched. Comparative studies by Kibar et al., Lee et al., and Behnke et al. show little difference between the groups [12, 14, 15]. Nearly all studies included a variety of fracture types according to the AO classification.

The meta-analysis revealed a significantly shorter operative time in the IMN group (50.8 ± 17.7 min) compared with the ORIF group (65.3 ± 28.7 min). This finding was consistent across all the included studies [10, 15–17]. Zhang et al. reported a mean operative time of 137 min for ORIF and 77 min for IMN. They attribute this to a smaller incision with less soft tissue dissection that may be particularly beneficial to cases that are time sensitive from a physiological standpoint.

The data suggest that IMN may have some advantages over ORIF. Nail fixation can be a suitable choice in patients with poor overlying skin that may result in infection or dehiscence requiring coverage [6, 12, 15]. Further, it is suggested that they may be of particular use in addressing highly comminuted or segmental fractures that are unlikely to be reduced anatomically with ORIF [12]. However, the efficacy of utilizing closed or minimal-access techniques to achieve adequate reduction and rotation are as of yet unclear. Some studies suggested that employing IMN as a less invasive yet rotationally stable construct carries high union rates and could reduce the risk of infection [14, 18–20].

A notable observation is the absence of refractures following the removal of intramedullary nails. Earlier studies have reported refracture rates after plate removal ranging from 5% to 20% [21, 22]. Removal of a nail does not necessitate repeat surgical dissection and does not leave as many areas of bone voids after screw removal. In addition, although scar cosmesis may be less of a concern to the orthopedic surgeon [23], Lee et al. reported lower satisfaction scores attributed to large scars in female patients receiving ORIF compared with IMN [15].

Although promising, use of IMN for forearm fractures is still relatively novel for most surgeons. The pre-contoured nails may require bending and additional contouring to match the variable patients’ native radial bow [24, 25]. Nails are unlikely to restore and maintain the anatomic bow as well as plates do, especially if closed reduction was performed [10, 13, 15]. However, residual angulation of less than 10° in any plane is unlikely to result in functional impairment [2]. Importantly, the concept of using nails for forearm fractures means that “relative stability” is achieved, defying the traditional dogma of the need for compression and anatomical reduction for the management of these fractures. Unfortunately, our review was unable to provide clear information on how many of the cases required some sort of an open incision to facilitate reduction. While general reporting regarding this was deficient, Saka et al. reported that 27% (16 out of 59 patients) required reduction via mini-incisions [26].

Hybrid fixation may provide some flexibility to surgeons in preoperative decision-making and seems to be an attractive tool. For instance, IMN can be utilized where the soft tissue envelope appears unfavorable [10, 12]. Zhang et al. found that the best results were achieved by nailing the ulna and plating the radius [10]. Further, plating still possesses the advantage of anatomical reduction in the radius. Although IMN are evolving and results are promising, surgeons should establish realistic expectations in line with the potential complications of using IMN in the forearm [27].

Our dataset underscores the need for caution to avoid recurrent complications. Notably, 11 instances of EPL ruptures were linked to IMN, necessitating reoperations for extensor pollicis longus reconstructions and tendon transfers, primarily diagnosed 2–6 weeks post-surgery. These injuries were often traced back to an ulnar entry point violating the third extensor compartment and creating bony spurs from Lister’s tubercle [12, 18, 28]. Thus, proper visualization, a more radial entry point, and hardware that is not prominent may mitigate this risk [12, 17, 18, 28]. Nerve palsies, impacting roughly 1% of IMN cases, were treated conservatively, though their precise cause remains elusive in most studies [10, 14, 19]. The IMN group saw a higher rate of delayed union at 3.1% versus 1% in ORIF, with the majority of reoperations addressing nonunions and EPL injuries.

This systematic review is subject to several limitations. Variations in study design, patient demographics, and the types of implants used across the included studies could potentially impact outcomes. A notable gap in the literature is the inconsistent reporting on postoperative protocols, rehabilitation, and institutional protocols, which were not controlled for in the studies. The interpretation of results is further complicated by the lack of standardized reporting on complications and follow-up durations. Additionally, the predominance of retrospective studies introduces potential biases.

Several factors may affect outcomes across the studies, including patient characteristics such as age, medical comorbidities, and osteoporosis. The recent introduction of the IMNs poses a limitation on the availability of long-term data. Furthermore, despite all implants sharing locking and anatomical features, there are limited data on how anatomical these nails truly are, whether they required contouring, and the stability each type achieves. Another area of concern is the nature of healing achieved; if nailing is performed without anatomical reduction and compression, it may result in secondary healing with callus formation. It would be valuable to explore whether the formation of a callus interferes with the range of motion.

The techniques employed by surgeons, particularly in terms of reduction methods (closed or open), were poorly reported and could influence infection rates and healing, especially if extensive soft tissue dissection occurred. Furthermore, a distinction in outcomes between closed reduction techniques and open techniques is necessary, as minimally invasive open techniques may have a higher likelihood of nerve injuries. In addition, studies should attempt to report the frequency of closed reduction attempts before converting to open reduction. Thus, complications for both approaches should be reported to determine whether closed reduction truly achieves proper reduction and rotational alignment.

As such, future research should scrutinize IMN outcomes across matched fracture types and severities. There would clearly be a learning curve associated with the technique, and determining techniques to address simple versus comminuted fractures is important. Furthermore, detailed descriptions of reduction techniques are warranted. It is crucial to discern whether any adjunctive measures, such as mini-incisions or open reductions, were employed alongside nailing. Furthermore, studies have largely overlooked the financial implications and cost-effectiveness of IMN compared with traditional plating methods.

## Conclusions

The use of anatomical interlocking IMN as an alternative to traditional ORIF with plates in the management of adult forearm diaphyseal fractures shows promise. Intramedullary nailing has demonstrated patient-reported outcomes comparable with ORIF along with shorter operative times and acceptable complication rates. While current studies indicate that IMN might be a safe alternative to ORIF, there is a need for higher quality research that matches fracture type and soft tissue conditions in controlled patients. Such research should focus on pinpointing appropriate indications, evaluating cost-effectiveness, and describing reduction techniques in detail to mitigate potential complications.

## Data Availability

The datasets used for this study are available by request from the Editor-in-chief.

## References

[1] Chung KC, Spilson SV (2001). The frequency and epidemiology of hand and forearm fractures in the United States. J Hand Surg Am.

[2] Dumont CE, Thalmann R, Macy JC (2002). The effect of rotational malunion of the radius and the ulna on supination and pronation. J Bone Jt Surg Br.

[3] Schulte LM, Meals CG, Neviaser RJ (2014). Management of adult diaphyseal both-bone forearm fractures. J Am Acad Orthop Surg.

[4] Patel A, Li L, Anand A (2014). Systematic review: Functional outcomes and complications of intramedullary nailing versus plate fixation for both-bone diaphyseal forearm fractures in children. Injury.

[5] Sage FP, Smith H (1957). Medullary fixation of forearm fractures. J Bone Jt Surg Am.

[6] Lee YH, Lee SK, Chung MS (2008). Interlocking contoured intramedullary nail fixation for selected diaphyseal fractures of the forearm in adults. J Bone Jt Surg Am.

[7] Stuck AE, Rubenstein LZ, Wieland D (1998). Bias in meta-analysis detected by a simple, graphical. BMJ.

[8] Duval S, Tweedie R (2000). A nonparametric, “trim and fill” method of accounting for publication bias in meta-analysis. J Am Stat Assoc.

[9] Rodgers MA, Pustejovsky JE (2021). Evaluating meta-analytic methods to detect selective reporting in the presence of dependent effect sizes. Psychol Method.

[10] Zhang XF, Huang JW, Mao HX (2016). Adult diaphyseal both-bone forearm fractures: a clinical and biomechanical comparison of four different fixations. Orthop Traumatol Surg Res.

[11] Pavone V, Ganci M, Papotto G (2021). Locked intramedullary nailing versus compression plating for stable ulna fractures: a comparative study. J Funct Morphol Kinesiol.

[12] Behnke NM, Redjal HR, Nguyen VT, Zinar DM (2012). Internal fixation of diaphyseal fractures of the forearm: a retrospective comparison of hybrid fixation versus dual plating. J Orthop Trauma.

[13] Kim SB, Heo YM, Yi JW (2015). Shaft fractures of both forearm bones: the outcomes of surgical treatment with plating only and combined plating and intramedullary nailing. Clin Orthop Surg.

[14] Kibar B, Kurtulmuş T (2020). Comparison of new design locked intramedullary nails and plate osteosynthesis in adult isolated diaphyseal radius fractures. Eur J Trauma Emerg Surg.

[15] Lee SK, Kim KJ, Lee JW, Choy WS (2014). Plate osteosynthesis versus intramedullary nailing for both forearm bones fractures. Eur J Orthop Surg Traumatol.

[16] Kibar B, Kurtulmuş T (2019). Treatment of adult isolated ulnar diaphyseal fractures: a comparison of new-generation locked intramedullary nail and plate fixation. Eklem Hastalik Cerrahisi.

[17] Kose A, Aydin A, Ezirmik N, Yildirim OS (2017). A comparison of the treatment results of open reduction internal fixation and intramedullary nailing in adult forearm diaphyseal fractures. Turkish J Trauma Emerg Surg.

[18] Blažević D, Benčić I, Ćuti T (2021). Intramedullary nailing of adult forearm fractures: results and complications. Injury.

[19] Kale SY, Singh SD, Samant P (2021). Treatment of diaphyseal forearm fracture with interlocking intramedullary nailing: a pilot study. J Clin Orthop Trauma.

[20] Azboy I, Demirtaş A, Alemdar C (2017). A newly designed intramedullary nail for the treatment of diaphyseal forearm fractures in adults. Ind J Orthop.

[21] Beaupré GS, Csongradi JJ (1996). Refracture risk after plate removal in the forearm. J Orthop Trauma.

[22] Anantavorasakul N, Lans J, Wolvetang NHA (2022). Forearm plate fixation: should plates be removed?. Arch Bone Jt Surg.

[23] Lari A, Alherz M, Hussain S (2022). The importance of scar cosmesis across the surgical specialties: factors, perceptions, and predispositions. Plast Reconstr Surg Glob Open.

[24] Gao H, Luo CF, Zhang CQ (2005). Internal fixation of diaphyseal fractures of the forearm by interlocking intramedullary nail: short-term results in eighteen patients. J Orthop Trauma.

[25] Weckbach A, Blattert TR, Ch W (2006). Interlocking nailing of forearm fractures. Arch Orthop Trauma Surg.

[26] Saka G, Saglam N, Kurtulmuş T (2014). New interlocking intramedullary radius and ulna nails for treating forearm diaphyseal fractures in adults: a retrospective study. Injury.

[27] Lari A, Alherz M, Jarragh A (2022). Dissociating advances in orthopaedic trauma management from the climbing patient expectations. Eur J Trauma Emerg Surg.

[28] Yörükoğlu AÇ, Demirkan AF, Akman A (2017). The effects of radial bowing and complications in intramedullary nail fixation of adult forearm fractures. Eklem Hastalik Cerrahisi.

[29] Weißer C, Weckbach A (2003). Interlocking nailing of forearm fractures with the foresight^™^ ulna/radius nail. Oper Orthop Traumatol.

[30] Visńa P, Beitl E, Pilný J (2008). Interlocking nailing of forearm fractures. Acta Chir Belg.

[31] Ozkaya U, Kiliç A, Ozdoğan U (2009). Comparison between locked intramedullary nailing and plate osteosynthesis in the management of adult forearm fractures. Acta Orthop Traumatol Turc.

[32] Bansal H (2011). Intramedullary fixation of forearm fractures with new locked nail. Indian J Orthop.

[33] Lil NA, Makkar DS, Aleem AA (2012). Results of closed intramedullary nailing using talwarkar square nail in adult forearm fractures. Malays Orthop J.

[34] Saka G, Sağlam N, Kurtulmuş T (2013). Interlocking intramedullary ulna nails in isolated ulna diaphyseal fractures: a retrospective study. Acta Orthop Traumatol Turc.

[35] Saka G, Saglam N, Kurtulmus T (2014). Treatment of isolated diaphyseal fractures of the radius with an intramedullary nail in adults. Eur J Orthop Surg Traumatol.

[36] Köse A, Aydın A, Ezirmik N (2014). Alternative treatment of forearm double fractures: new design intramedullary nail. Arch Orthop Trauma Surg.

[37] Al-Sadek TA, Niklev D, Al-Sadek A (2016). Diaphyseal fractures of the forearm in adults, plating or intramedullary nailing is a better option for the treatment?. Open Access Maced J Med Sci.

[38] Köse A, Aydın A, Ezirmik N (2016). Intramedullary nailing of adult isolated diaphyseal radius fractures. Ulus Travma Acil Cerrahi Derg.

[39] Babu PA, Rao VVN (2017). Diaphyseal fractures of ulna and radius with soft tissue breach-our experience with square nail fixation. J Evolut Med Dental Sci Jemds.

[40] Lee SK, Kim YH, Kim SM, Choy WS (2019). A comparative study of three different surgical methods for both-forearm-bone fractures in adults. Acta Orthop Belg.

[41] Uygur E, Özkut A, Akpınar F (2021). Synostosis after fracture of both forearm bones treated by intramedullary nailing. Hand Surg Rehabil.

